# Heterologous Expression of Full-Length and Truncated Human ZIP4 Zinc Transporter in *Saccharomyces cerevisiae*

**DOI:** 10.3390/biom12050726

**Published:** 2022-05-21

**Authors:** Yuting Liu, Elizabeth M. Bafaro, Robert E. Dempski

**Affiliations:** Department of Chemistry and Biochemistry, Worcester Polytechnic Institute, Worcester, MA 01609, USA; yliu23@wpi.edu (Y.L.); ebafaro@wpi.edu (E.M.B.)

**Keywords:** human zinc transporter, membrane protein, heterologous expression in yeast, GFP fusion protein

## Abstract

The human (h) transporter hZIP4 is the primary Zn^2+^ importer in the intestine. hZIP4 is also expressed in a variety of organs such as the pancreas and brain. Dysfunction of hZIP4 can result in the Zn^2+^ deficiency disease acrodermatitis enteropathica (AE). AE can disrupt digestive and immune system homeostasis. A limited number of hZIP4 expression strategies have hindered increasing knowledge about this essential transmembrane protein. Here, we report the heterologous expression of hZIP4 in *Saccharomyces cerevisiae*. Both a wild-type and a mutant *S. cerevisiae* strain, in which the endogenous Zn^2+^ transporters were deleted, were used to test the expression and localization of an hZIP4–GFP fusion protein. A full-length hZIP4–GFP and a truncated membrane-domain-only (mhZIP4–GFP) protein were observed to be present in the plasma membrane in yeast.

## 1. Introduction

Zn^2+^ is an essential nutrient for human health. For example, Zn^2+^ functions as a catalytic and structural cofactor for Zn^2+^-dependent enzymes and transcription factors. Zn^2+^ also contributes to cell signaling [1,2,3]. Befitting its central importance to human health, Zn^2+^ deficiency can lead to immune system dysfunction, growth retardation, and neurological disorders [4]. While Zn^2+^ deficiency is primarily caused by inadequate dietary intake of Zn^2+^ [5], genetic factors can also influence human Zn^2+^ levels.

Two classes of Zn^2+^ transporters regulate intracellular Zn^2+^ levels. ZIP (for Zrt-, Irt-like Protein) transporters comprise the solute-linked carrier (SLC)39 family and function to increase cytosolic Zn^2+^ levels. The SLC30 family ZnT (for Zn^2+^ Transporter) proteins function to decrease cyosolic Zn^2+^ levels [6]. hZIP4 is expressed in a variety of cell types including the intestine, the primary location of Zn^2+^ uptake, as well as the pancreas [7,8]. Overexpression of hZIP4 has been shown to increase the expression of proteins that can initiate or progress pancreatic cancer [9]. Mutations in hZIP4 can lead to the Zn^2+^ deficiency disease acrodermatitis enteropathica (AE) [10,11]. AE is an autosomal recessive genetic disorder that can lead to death if left untreated. Important for the rationale of the work described here, previous studies have shown that the N-termini of the mouse (m) ZIP4 protein is removed during prolonged Zn^2+^ deficiency growth condition [12]. hZIP4 protein lacking the N-terminal extracellular domain was also observed in response to Zn^2+^ deficiency in epithelial CaCo-2 and Hepa cells [13,14]. This provides further evidence that the N-terminus of hZIP4 is physiologically relevant when cells are Zn^2+^ deficient. 

Previously, expression systems used to overexpress human ZIP proteins include mammalian cultured cells and *Xenopus laevis* oocytes. For example, hZIP1 and hZIP2 were shown to be functionally expressed in PC-3 cells and K562 cells, respectively [15,16]. In addition, previous studies examined the functionality of hZIP4 following expression in *Xenopus laevis* oocyte and HEK293 cells [17,18,19]. Moreover, the impact of ZIP4 overexpression on cancer cell formation was studied using human pancreatic cancer cell lines [7]. However, using cultured human cells or *Xenopus laevis* oocytes has limited utility due to high cost when compared to yeast expression systems. While the sole soluble domain of hZIP4 has been purified following overexpression in *E. coli*, this is not a reliable strategy for the full-length mammalian transporter [20]. In addition, yeast offers a rapid cloning mechanism, and its genome has been well studied for years. Furthermore, different strains encode metal transporter deletions or are knocked out and provide an alternative platform for heterologous protein expression. This could be of importance, as no structure has been elucidated for hZIP proteins. 

**Figure 1:**
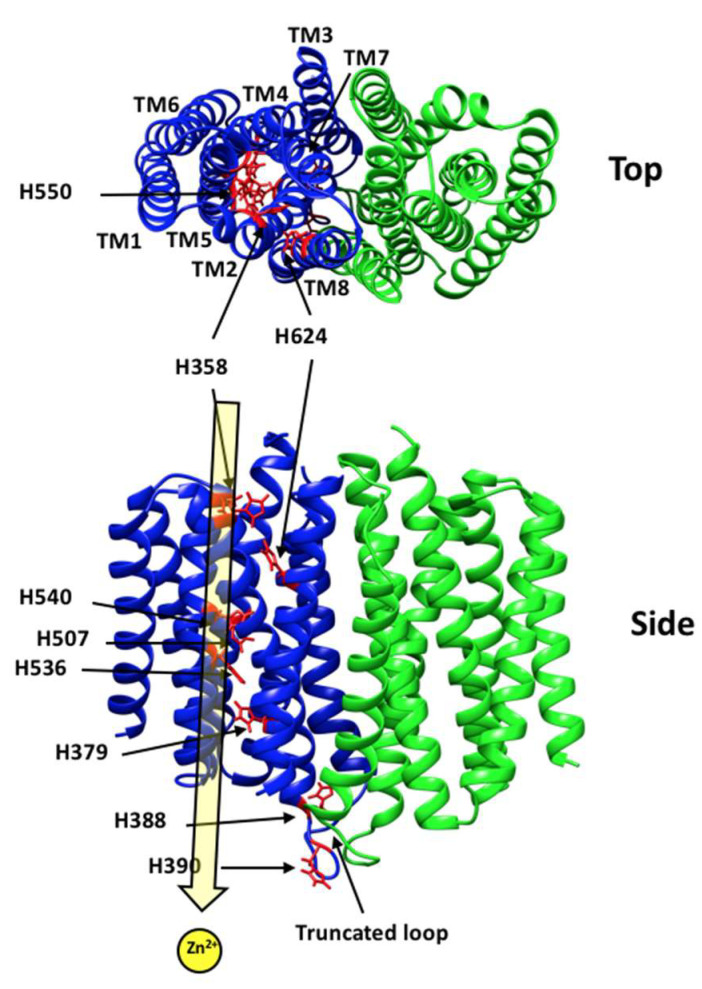
hZIP4 transmembrane homodimer view. Computational hZIP4 structure generated by Rosetta ab initio structure with co-evolution contact prediction [17]. The predicted locations of the transmembrane histidine residues are displayed in red. The putative Zn^2+^ translocation pore is highlighted in yellow. TM domains are numbered.

Our current understanding of the mechanism of hZIP4 comes from functional studies in human cultured cells, mice, and computational approaches [11,17,21,22,23]. Functional studies indicate that hZIP4 can translocate Zn^2+^, Cu^2+^, Ni^2+^, and Fe^2+^ [17,23]. Studies in mice showed that expression of mouse (m) ZIP4, which shares 75% sequence similarity to hZIP4, is regulated by dietary Zn^2+^ levels [8]. When cellular Zn^2+^ levels are high, mZIP4 is endocytosed in cultured cells [24]. In hZIP4, the sole significant intracellular domain encodes a histidine-rich region. It was shown that these histidines sense intracellular Zn^2+^ levels. When Zn^2+^ levels are high, hZIP4 is ubiquitinated and targeted for degradation [22]. This domain is disordered and coordinates 2 Zn^2+^ sequentially [20,25]. An ab initio modelling study provided the first structural insight into the transmembrane helices of hZIP4 (Figure 1) [17]. Direct structural information on the transmembrane domains of hZIP4 is not yet available; however, elucidation of the crystal structure of a bacterial ZIP homology has provided a template for modeling efforts [26]. Both the ab initio model and bacterial ZIP homolog structure illustrate eight transmembrane helices, with conserved histidine and aspartate residues lining the putative metal transport pathway (Figure 1).

*Saccharomyces cerevisiae* is a robust, single-cell eukaryotic model system that can be used for heterologously expressed transmembrane proteins. The advantages of *S. cerevisiae* include a fully sequenced genome, genetic tractability using classical genetic approaches, and the ability to grow on chemically defined media [27]. As a model organism for transition metal expression, the genes involved in *S. cerevisiae* metal transport have been identified, many of which are homologous to metal transporter genes from other organisms including humans [27]. A variety of yeast-transition–metal-transport-deficient strains have been generated and used to characterize heterologously expressed metal transporters, including Fe^2+^ [28], Cu^+/2+^ [29], and Zn^2+^ [30].

As it is often difficult to heterologously express eukaryotic membrane proteins for high-resolution biochemical and structural studies, *S. cerevisiae* has been explored as a host organism to overexpress membrane proteins using GFP-based fusion technology. As the C-terminal GFP folds and becomes fluorescent only when the upstream membrane protein integrates into the membrane, the resultant fluorescence is a fast and accurate measure of membrane-integrated expression [31]. Our approach here takes advantage of a reliable, high-throughput protocol for overexpression and screening of eukaryotic membrane proteins in *S. cerevisiae* [32]. Confocal microscopy analysis of several eukaryotic membrane protein–GFP fusions under overexpression conditions demonstrated targeting of the overexpressed membrane proteins to the correct organelle in *S. cerevisiae*. Through the adjustment of the protein-inducing conditions, maximal target protein levels can be obtained for further purification studies, and the membrane protein–GFP fusion can be screened by fluorescence size-exclusion chromatography [33]. While the human ZIP1 full-length transporter has been expressed, our objective within the work described here was to express a full-length and truncated version of hZIP4, as this protein is expressed in the plasma membrane of the intestine, the main location of Zn^2+^ update [34]. In this study, we successfully expressed hZIP4 tagged with green fluorescent protein (GFP) in *S. cerevisiae* and show that the hZIP4–GFP fusion protein is targeted to the yeast plasma membrane.

## 2. Materials and Methods

### 2.1. Yeast Strains, Plasmids, Media, and Reagents

The *S. cerevisiae* strains used in this study were kindly provided by the Eide Lab (University of Wisconsin-Madison, Madison, WI, USA): wild-type strain DY1457 (MATα, *ade6*, *can1*, *ura3*, *leu2*, *his3*, *trp1*) and Zn^2+^-transport-deficient strain ZHY3 Δ*zrt1*/Δ*zrt2* (MATα, *ade6*, *can1*, *his3*, *leu2*, *trp1*, *ura3*, *zrt1::LEU2*, *zrt2::HIS3*). The plasmid pDDGFP-LEU2D was a gift from Simon Newstead (Addgene plasmid # 58352). The plasmid pPICZ was used to obtain the yeast α-factor secretion signal sequence and was obtained from ThermoFisher Scientific (Waltham, MA, USA). For the culture media, Yeast Peptone Dextrose (YPD) contained 1% (*w*/*v*) yeast extract, 2% (*w*/*v*) peptone, and 2% (*w*/*v*) glucose, which were purchased from Bacto^TM^, HIMEDIA, and Sigma, respectively. Synthetic Defined Culture (SDC) medium was made with 1.7% (*w*/*v*) Zn^2+^-free Yeast Nitrogen Base (YNB-ZnSO_4_) (Sunrise Science Products), 5% (*w*/*v*) (NH_4_)_2_SO_4_ (VWR), amino acid supplements without uracil (-URA) (US Biological Life Sciences), and 2% (*w*/*v*) glucose (non-inducing) or galactose (inducing) (Sigma), and then the media were adjusted to pH 4.2 with 10 mM citric-Na_3_Citrate buffer (Fisher Scientific). Trace metal supplements, MnCl_2_ and FeCl_3_, were purchased from Alfa Aesar, and EDTA was obtained from Fisher Scientific. For confocal microscopy, Yeast Suspension Buffer (YSB) was used to slow the cells mobility; YSB was made of 5 mM EDTA, 50 mM Tris-HCl pH 7.6, and 10% (*v*/*v*) glycerol.

### 2.2. Plasmid Construction

Two hZIP4 gene constructs were inserted into the yeast vector pDDGFP-LEU2D [35]: the full-length hZIP4 gene (residues 1–647) and a truncated construct (mhZIP4) encoding only the eight transmembrane segments (residues 328–647). The hZIP4 genes were preceded by the yeast α-factor secretion signal sequence (Table 1) to enhance processing of the gene product to the plasma membrane. The genes were cloned into the pDDGFP-LEU2D vector, which encodes a C-terminal GFP, by homologous recombination in yeast cells that were transformed with the DNA insert and plasmid using the lithium acetate procedure [36]. Following insertion into the pDDGFP-LEU2D plasmid, the hZIP4 and mhZIP4 genes encoded a fusion protein with an N-terminal α-factor secretion signal and a C-terminal GFP under the control of a *gal1* promoter [36]. As a result, protein induction was triggered by replaced the liquid media to 2% galactose SDC media. Yeast colonies were selected on SDC plates incubated at 30 for 3 days. Plasmid sequences were confirmed by sequencing the entire gene.

### 2.3. S. cerevisiae hZIP4 Protein Expression and Localization

The expression of hZIP4 or mhZIP4 protein in *S. cerevisiae* wild type and Zn^2+^-deficient strains was assessed by quantifying the amount of GFP fluorescence after hZIP4 or mhZIP4 expression was induced by 2% (*w*/*v*) galactose as previously described [36]. Yeast cells transformed with the empty vector were used as the control. After induction for 22–24 h, cells were harvested at 3000× *g* for 5 min, washed, and resuspended in YSB to an OD_600_ of 6. GFP fluorescence was measured using a PerkinElmer VICTOR^3^ Multilabel Counter using an excitation wavelength of 488 nm and an emission wavelength of 512 nm with the microplate set to bottom read [36]. For confocal microscopy, induced cells were resuspended in YSB, and a drop of cell culture was spotted on a 1% agar pad (made with SDC medium) on a glass slide. The agar pad was sealed with VALAP (1:1:1 parts of Vaseline, lanoline, and paraffin) by cover slip [37]. Samples were focused with transmitted light at 10× magnification, then switched to blue light to estimate the gross localization of GFP. Laser scanning confocal microscopy was performed on a Leica TCS SP5 confocal microscope. Laser beams with 488 nm excitation and 503–530 nm emission wavelengths were used for GFP. Single confocal sections and z-stack images were processed in ImageJ [36,38].

### 2.4. Measurement of Growth Curves

Yeast cells were transformed, grown, and harvested as above, then switched to 2% (*w*/*v*) glucose or 2% (*w*/*v*) galactose SDC for measuring the growth under non-inducing condition or inducing condition, respectively. Two Zn^2+^ concentrations (2 mM and 0.5 mM) were applied to each condition. The liquid culture was incubated at 30 °C with 220 rpm shaking for 30 h or 120 h starting at an initial OD_600_ of 0.1. Cell growth was monitored by measuring OD_600_ as a function of time.

## 3. Results

### 3.1. hZIP4 Was Heterologously Expressed in S. cerevisiae and Localized to the Plasma Membrane

**Figure 2:**
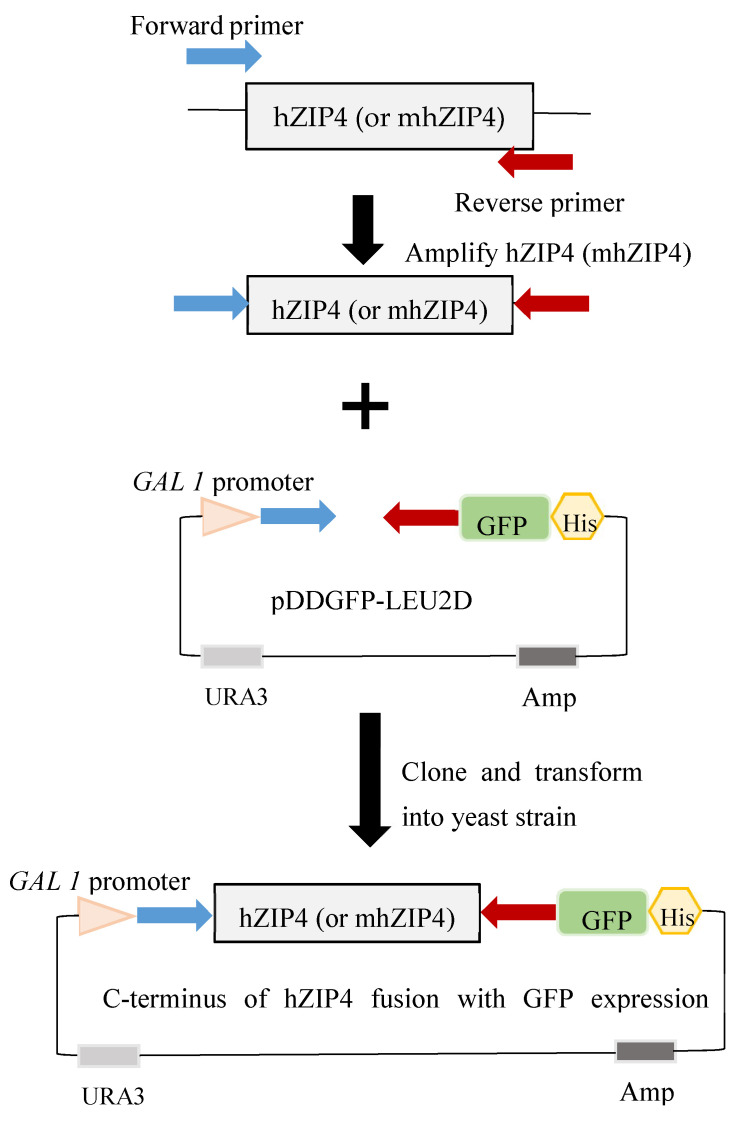

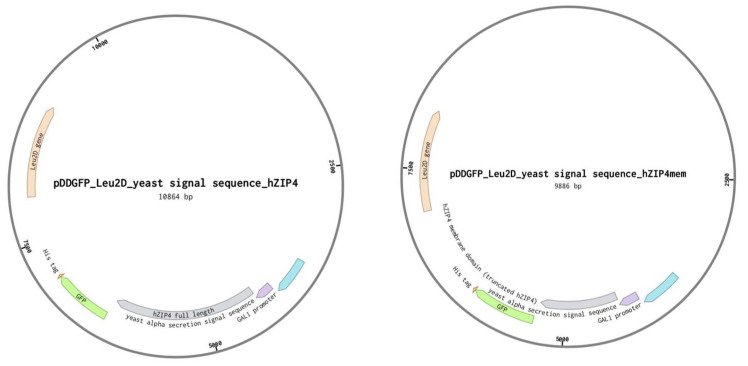
(**Top**) hZIP4-GFP and mhZIP4-GFP plasmid construction. GFP was fused to C-terminal of hZIP4 and mhZIP4 plasmids, respectively. hZIP4 or mhZIP4 was amplified and inserted into pDDGFP-LEU2D vector with C-terminal fusion GFP as indicator. The expression of hZIP4 or mhZIP4 was induced by galactose. (**Bottom**) Plasmid maps of hZIP4 and mhZIP4. The full length of hZIP4 and mhZIP4 was cloned into pDDGFP-LEU2D vector with GFP at C-terminal. *GAL1* promoter allowed protein expression induced by galactose.

**Figure 3:**
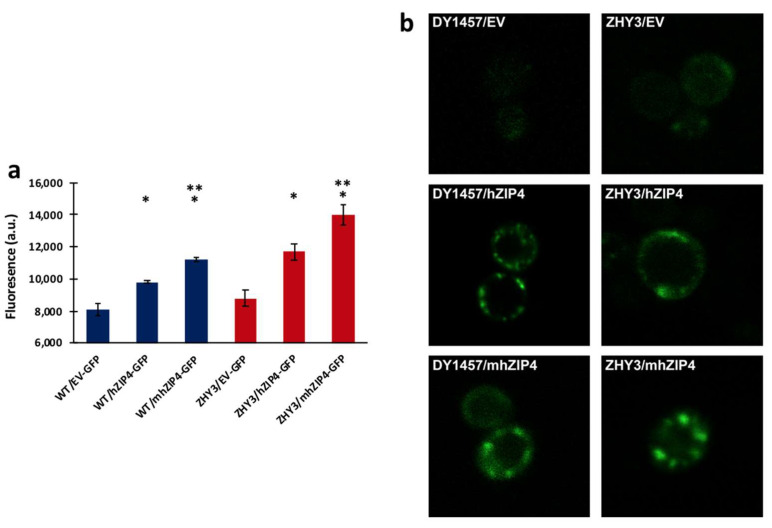
hZIP4 and mhZIP4 expression in *Saccharomyces cerevisiae* wild-type strain (WT) DY1457 and Zn^2+^-transport-deficient strain (ZHY3). (**a**) Fluorescence measurements following 12-hour protein induction. GFP fluorescent levels are indicated by fluorescence in arbitrary units (a.u.). The background fluorescence was measured using cells transformed with the yeast empty vector (EV) pDDGFP-LEU2D. Data represent the average ± SD (*n* = 9). * indicates statistically significant increase in fluorescence for hZIP4 or mhZIP4 when compared to empty vector (EV) control. ** indicates statistically significant difference in fluorescence for mhZIP4 when compared to hZIP4 (*t* test: *p*-value < 0.05). (**b**) Cellular localization of hZIP4 and mhZIP4 expressed in WT and ZHY3. All the transformed cells were 22-hour induced with galactose before visualization by confocal microscopy. Cells transformed with the empty vector (EV) are shown as the control.

GFP fused to the C-terminus of hZIP4 was used as a reporter to monitor expression and localization of hZIP4 or the truncated mhZIP4 (hZIP4 membrane domain only) (Figure 2). hZIP4–GFP (or mhZIP4–GFP) protein expression was induced upon addition of 2% (*w*/*v*) galactose. GFP fluorescence was measured in wild type (DY1457) and ZHY3 (Zn^2+^ transport deficient strain, Δzrt1/Δzrt2). Background fluorescence values were taken using the same conditions, except that cells were transformed with an empty vector as described here [36]. In both wild-type and ZHY3 cells, hZIP4-GFP and mhZIP4-GFP were expressed as indicated by the increase in fluorescence compared to the background empty vector controls (Figure 3a). Expression levels in the Zn^2+^-transporter-deficient ZHY3 strain were twice as high as in the wild-type DY1457 strain. Additionally, the truncated, membrane domain hZIP4–GFP fusion expression was twofold higher than the full-length hZIP4–GFP, regardless of the strain.

The cellular localization of hZIP4–GFP and mhZIP4–GFP was assessed using confocal microscopy (Figure 3b). As expected, the fluorescence levels were low for both yeast strains when transformed with the empty vector. In contrast, congruent with the total fluorescence levels observed (Figure 3a), an increase in fluorescence was observed for hZIP4–GFP and mhZIP4–GFP in both strains. Here, fluorescent rings on the periphery of the cells indicated localization of the proteins to the plasma membrane of the yeast cells. In cells expressing higher protein levels, some fluorescent protein accumulated in subregions of the plasma membrane. This punctuated distribution pattern has been observed for other proteins that localize in microdomains within the plasma membrane [39]. Considering that fluorescence originating from the C-terminal GFP will only be observed if the full-length protein is folded and that the protein is accumulated in the plasma membrane region, this is supportive of the idea that the full-length proteins have been expressed. Together, these results demonstrate that hZIP4–GFP and mhZIP4–GFP can be heterologously expressed and then relocate to the surface of S. cerevisiae.

### 3.2. Role of hZIP4 in Growth Rate Control

**Figure 4:**
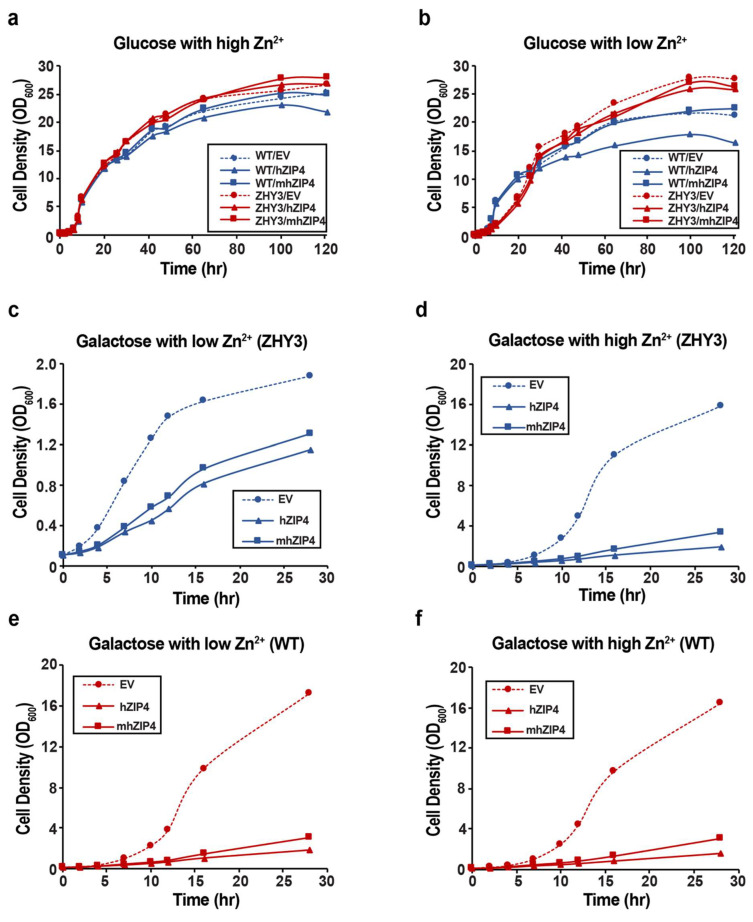
Growth curve of ZHY3 and WT transformed with hZIP4 and mhZIP4. All cells were cultured in synthetic defined culture (SDC) with glucose overnight. Cells were diluted to OD600 0.1 in SDC with glucose (non-inducing) or galactose (inducing) under Zn^2+^-deficient (0.5 mM) and Zn^2+^-replete (2 mM) conditions. (**a**,**b**) ZHY3 and WT with hZIP4, mhZIP4, and EV in glucose. (**c**,**d**) ZHY3 with hZIP4, mhZIP4, and EV in galactose. (**e**,**f**) WT with hZIP4, mhZIP4, and EV in galactose. Experiments were measured twice independently.

To assess the impact of hZIP4–GFP and mhZIP4–GFP expressed in S. cerevisiae, growth curves were measured with ZHY3 and wild-type strains. Both strains were transformed with the yeast empty vector (EV), hZIP4–GFP, or mhZIP4–GFP. Growth curves were obtained in liquid media under non-inducing (glucose) or inducing (galactose) conditions with high (2 mM) or low (0.5 mM) Zn^2+^ (media was chelex-treated by adding 1 mM EDTA). Under the non-inducing condition, with high Zn^2+^ levels, growth curves for ZHY3 or wild type transformed with the empty vector, and hZIP4–GFP and mhZIP4–GFP plasmids were indistinguishable (Figure 4a). For both cell types, the lag phases were identical, and, during exponential growth, the doubling times (T_D_) were less than two hours. The cells under these conditions looked healthy, while increases in fluorescence were observed. When the same cells were grown under Zn^2+^-limiting conditions, differences between the wild-type and Zn^2+^-transport-deficient mutant ZHY3 strains were observed (Figure 4b). In Zn^2+^-deficient media, the growth curves of wild-type strain transformed with the empty vector, and hZIP4–GFP and mhZIP4–GFP plasmids were similar to those seen in Zn^2+^ replete media. However, for the ZHY3 strain transformed with the empty vector, hZIP4–GFP or mhZIP4–GFP plasmids, the exponential growth rate in Zn^2+^-deficient media was significantly slower compared to the wild-type strain (Figure 4b). This result was expected as the ZHY3 strain is missing both Zn^2+^ importers and grows slowly in Zn^2+^-deficient media [40]. Quantitatively, T_D_ for each of the transformed wild-type cells remained at two hours, whereas T_D_ for each of the transformed ZHY3 cells was more than six hours in Zn^2+^-limiting media. 

In contrast to non-inducing conditions where growth curves were similar for all variants, significant differences in growth curves were observed for ZHY3 and wild-type cells upon hZIP4 or mhZIP4 protein expression when compared to the empty vector control, under inducing (galactose) conditions (Figure 4c–f). In the presence of high levels of Zn^2+^ (Figure 4d,f), growth curves for the ZHY3 and wild type expressed with empty vector were similar to those seen in non-inducing conditions. In contrast, induction of hZIP4 or mhZIP4–GFP for both strains resulted in cell growth that was significantly slower, and it did not reach the exponential phase, compared to the cells transformed with the empty vector. Expression of hZIP4–GFP in both wild-type and ZHY3 resulted in an even slower lag phase than mhZIP4–GFP-expressing cells in inducing conditions and in Zn^2+^ replete media. In the presence of protein-inducing conditions and Zn^2+^-limited media (0.5 mM Zn^2+^), wild-type cells transformed with the empty vector and grew significantly faster than the ZHY3 strain (Figure 4c,e). Again, it was observed that heterologous expression of mhZIP4–GFP resulted in a significantly slower growth rate for both wild-type (DY1457) and ZHY3 cells when compared to the empty vector (Figure 4c,e). In addition, heterologous expression of hZIP4–GFP resulted in a larger decrease in cell growth when compared to cells transformed with mhZIP4–GFP.

## 4. Discussion

hZIP4 is a plasma membrane protein expressed in various cell types including the intestine and pancreas [7,8]. Mutations in hZIP4 lead to the lethal genetic disorder acrodermatitis enteropathica [41]. Therefore, maintenance of Zn^2+^ homeostasis is essential for human health. Studies of ZIP4 expressed in mice, cultured cells, and *X. laevis* oocytes have provided useful insights into the regulation of hZIP4, its metal substrate specificity, and predicted metal transport pathway [8,17,23,24]. *S. cerevisiae* provides an attractive alternative for heterologous expression of metal transporters because the metal homeostatic mechanisms are well defined in yeast [27]. We report here the first yeast-based heterologous expression system for full-length and truncated hZIP4. Two hZIP4–GFP fusions, one for the full-length transporter and one for the membrane domain only (mhZIP4), were expressed in wild-type (DY1457) and Zn^2+^-transport-deficient (ZHY3) *S. cerevisiae* strains. GFP fluorescence levels and confocal microscopy confirmed the successful heterologous expression and plasma membrane localization of the hZIP4–GFP and mhZIP4–GFP fusion proteins.

The ZIP family of metal transporters has been shown to increase cytosolic Zn^2+^ levels in vivo. Deletion of the two ZIP genes, *zrt1* and *zrt2*, in *S. cerevisiae* produces a growth phenotype sensitive to Zn^2+^-limited conditions. Thus, functional expression of hZIP4 in the *zrt1*/*zrt2* deletion strain *S. cerevisiae* ZHY3 was expected to restore its Zn^2+^-dependent growth rate, as has been shown for a number of plant and fungal ZIP homologues that have been expressed in ZHY3 [42,43,44]. Surprisingly, under our experimental conditions, growth of ZHY3 expressing either full-length hZIP4 or the hZIP4 membrane domain was initially similar, but later in the time course repressed in Zn^2+^-limited growth conditions. Similarly, growth of the wild-type strain *S. cerevisiae* DY1457, which has normal growth on both Zn^2+^-replete and Zn^2+^-deficient media, was repressed upon induction of hZIP4–GFP or mhZIP4–GFP protein expression. The repressed growth rates observed upon expression of hZIP4–GFP or mhZIP4–GFP could have been due to an increased metabolic burden associated with heterologous membrane protein overexpression or toxicity of the expressed protein in yeast.

Another interesting result derived from our yeast-based hZIP4 expression system is that the truncated, membrane domain of hZIP4 was expressed and targeted to the yeast plasma membrane and had the same effect on cell growth rate compared to the full-length hZIP4. The large N-terminal ectodomain is cleaved under extended Zn^2+^ deficiency, and mutations in the N-terminus have been identified in acrodermatitis enteropathica cases [14]. The study by Kambe and Andrews demonstrated that the processed mouse ZIP4, consisting of the membrane domain after ectodomain cleavage, was functional for Zn^2+^ uptake in mouse and human cells [14]. However, a more recent study in HEK293T cells showed that the truncated hZIP4 was significantly impaired for Zn^2+^ uptake [45]. Our heterologous, yeast-based expression system is advantageous over mammalian cell lines because the yeast system provides a well-defined metal transport background. *S. cerevisiae* encodes two Zn^2+^ transporters, Zrt1 and Zrt2. Both of these transporters are deleted in the ZHY3 strain. In addition, the yeast-based system has the advantage of overexpressing heterologous membrane protein in large scale and can be easily monitored using GFP levels and confocal microscopy, since the C-terminal GFP folds and becomes fluorescent only if the upstream membrane protein integrates into the membrane. The yeast-based hZIP4 expression system developed here could be a valuable platform to heterologously overexpress integral membrane metal transporter proteins.

Among the advantages of using a yeast model system for membrane protein expression, recent studies have shown that *S. cerevisiae* is flexible in high-throughput fluorescent-based eukaryotic membrane protein overexpression [32], and yeast is considered to be a facile platform for high-throughput screening of protein inhibitors or activators [46]. Although yeast-based screening may not be as accurate as mammalian cells due to differences in post-translational modification and other physiological differences, yeast-based assays are more facile for high-throughput screening by simply monitoring cell growth. Currently, there are no known drugs that target the hZIP4 transporter, and the yeast-based expression for hZIP4 described here may provide a useful tool for screening molecules that activate or inhibit the target hZIP4 protein.

## Figures and Tables

**Table 1:** Primer sequences. Full-length and truncated hZIP4 primer sequences. Primers of hZIP4 and mhZIP4 *S. cere* signal sequence.

Sequences	Primer Sequence (5′-3′)
hZIP4 *S. cere* signal sequence forward	ACCCCGGATTCTAGAACTAGTGGATCCCCCATGAGATTTCCTTCAATTTTTACTGC
hZIP4 full-length overlap extension forward	GAGAGGCTGAAGCTTACGTAGCGTCCCTGGTCTCGCTGGAGC
hZIP4 full-length overlap extension reverse	GCTCCAGCGAGACCAGGGACGCTACGTAAGCTTCAGCCTCTC
hZIP4 *S. cere* signal sequence reverse	AAATTGACCTTGAAAATATAAATTTTCCCCAGAACCACCGAAGGTGATGTCATCCTCGTAC
Truncated hZIP4 *S. cere* signal sequence forward	ACCCCGGATTCTAGAACTAGTGGATCCCCCATGAGATTTCCTTCAATTTTTACTGC
Truncated hZIP4 full-length overlap extension forward	GAGAGGCTGAAGCTTACGTACTGTACGGCTCCCTGGCCACGC
Truncated hZIP4 full-length overlap extension reverse	GCGTGGCCAGGGAGCCGTACAGTACGTAAGCTTCAGCCTCTC
Truncated hZIP4 *S. cere* signal sequence reverse	AAATTGACCTTGAAAATATAAATTTTCCCCAGAACCACCGAAGGTGATGTCATCCTCGTAC

## Data Availability

Data is available upon request.
